# Implementation of flipped classroom combined with case-based learning: A promising and effective teaching modality in undergraduate pathology education

**DOI:** 10.1097/MD.0000000000028782

**Published:** 2022-02-04

**Authors:** Li Cai, Yan-li Li, Xiang-yang Hu, Rong Li

**Affiliations:** a Department of Pathology, School of Basic Medicine, Anhui Medical University, Hefei, Anhui Province, P.R. China; b Inflammation and Immune Mediated Diseases Laboratory of Anhui Province, School of Pharmacy, Anhui Medical University, Hefei, Anhui Province, P.R. China.

**Keywords:** flipped classroom, medical education, pathology, teaching reform, traditional lecture-based classroom

## Abstract

The popularity of flipped classroom (FC) is growing in medical education. However, the application of FC in pathology teaching has not been well explored. This study assessed the efficacy of FC combined with case-based learning (CBL) in undergraduate pathology education via comparison with a traditional lecture-based classroom (LBC).

A total of 117 third-year students were enrolled and assigned to the FC group (n = 59) or LBC group (n = 58) with demographic matches. Two sections in the pathology textbook (cardiovascular and respiratory system diseases) were chosen for the teaching content. Students in the FC group were required to study the preprovided course materials pre-class, followed by clinical case-based interactive group discussion in-class. Students in the LBC group were encouraged to preview and attended a didactic lecture in class. Post-class quizzes and Likert questionnaires were performed to investigate the efficacy and possible advantages of CBL-based FC over LBC.

The scores of the 2 groups in the mid-term examination of pathology before interventions were comparable. However, students in the FC group gained significantly higher scores in the post-quizzes than those in the LBC group, especially the scores regarding the questions of clinical case analysis. In the questionnaires, more students considered CBL-based FC to be beneficial to learning motivation, knowledge comprehension, critical thinking, patient management and teamwork than LBC. In addition, more students agreed that the FC model increased pre-class burden than LBC, rather than in-class pressure.

CBL-based FC modality has promising effects on undergraduate pathology education and may be a better choice than traditional LBC. Further optimizations are needed to implement this novel approach in pathology and other medicine curricula.

## 1 Introduction

As a bridge subject connecting basic medicine and clinical medicine, pathology is a compulsory course for worldwide medical students.^[1]^ The main goal of undergraduate pathology teaching is to provide students with an understanding of the functional and structural changes of disease, so that they can understand and interpret clinical signs and symptoms.^[2]^ Therefore, pathology teaching is central to the understanding of disease and is important to the medical education of physicians.^[3]^ Pathology teaching itself is a hard, complicated and challenging task, often with frustration.^[4]^ For years, pathology has developed from a macroscopy and autopsy based discipline to a finessed histological and molecular field with great advances. The growing advances in pathology but relatively lagging teaching models of pathology bring great challenges to pathology educators.^[5]^

Presently, the main teaching model in medicine curricula, including pathology in China, is still the traditional approach characterized by lecture-based classroom (LBC) and students’ in-class listening.^[6]^ In LBC, instructors deliver knowledge and concepts in a teacher-centered manner, and students collectively listen, take notes and passively study without understanding.^[7]^ Although this approach aimed at knowledge infusion helps to the memorization of basic knowledge in a limited period, many shortcomings have been found in the cultivation of the abilities of problem-solving, critical thinking, teamwork and self-active learning.^[8]^ Based on our experiences and previous reports,^[1,3,4]^ many medical students complained that the LBC-based pathology course was boring, and it was arduous to effectively learn this course. Given that medicine, including pathology, is a practical science, the LBC model cannot fulfill the requirements of the present medical education system and has been proven to be poorly effective in high-order learning abilities.^[9]^ Thus, innovative and modern teaching methods should be applied in pathology education to promote students’ abilities to solve real clinical problems.^[10]^

The flipped classroom (FC) is a brand-new pedagogical approach that inverts teacher-centered and lecture-based traditional education into student-centered active learning education.^[11]^ In FC progress, students study preprepared course materials pre-class without the restrictions of time and place, and participate in face-to-face interactive learning and problem solving in class, often with collaborative small group activities under the instructor’ guidance.^[12]^ FC leads to a shift from passive learning to active learning, facilitates higher order learning of the materials and promotes the development of various cardinal skills, thus overcoming the shortcomings of traditional LBC with desired results.^[13]^ The popularity of FC modality is growing in education, especially in various fields of medical education, such as anatomy, pharmacology, physiology, dermatology, and radiology.^[13–15]^ Meanwhile, case-based learning (CBL) is an in-class activity that can be applied within the FC model,^[16,17]^ where medical students work in groups to deal with questions related to disease diagnosis and clinical decision-making. However, the implementation of FC in pathology teaching has not been well explored. In this study, we administered FC combined with CBL in undergraduate pathology teaching to investigate the efficacy and potential advantages of this teaching model compared with the traditional LBC model, so as to provide evidence for the reform of pedagogical approaches in pathology education.

## 2 Materials and methods

### 2.1 Participants

This study was conducted at Anhui Medical University (Hefei, Anhui Province, China) in November 2019 (2019/2020 academic year), and a total of 117 third-year students majoring in clinical medicine were enrolled here. In their first 2 years of study, the students had completed Human Anatomy, Histology-Embryology, Human Physiology, Biochemistry, Molecular Biology, Medical Immunology, Medical Microbiology, Cell Biology and Human Parasitology courses, and mastered the basic knowledge of the subjects of fundamental medicine. Here, the pathology curriculum consists of 2 parts. The first part interprets the principles of general pathology, including cell injury, cell death and adaptations, tissue repair, hemodynamic disorders, inflammation, and neoplasia. The second part proceeds to specific disease processes as they affect particular organs or systems. The students who had not previously participated in inverted classroom had learned the first part of the pathology curriculum, and entered the mid-term examination of pathology to assess their previous performance on pathology learning. Participants were assigned to 2 groups: the FC group, wherein students received FC combined with CBL approach (n = 59); and the LBC group, wherein students received the traditional LBC approach (n = 58). This study was approved by the Institutional Review Board and Ethics Committee of Anhui Medical University (20190197), and all participants submitted their informed consent before this study.

### 2.2 Study design

**Figure 1:**
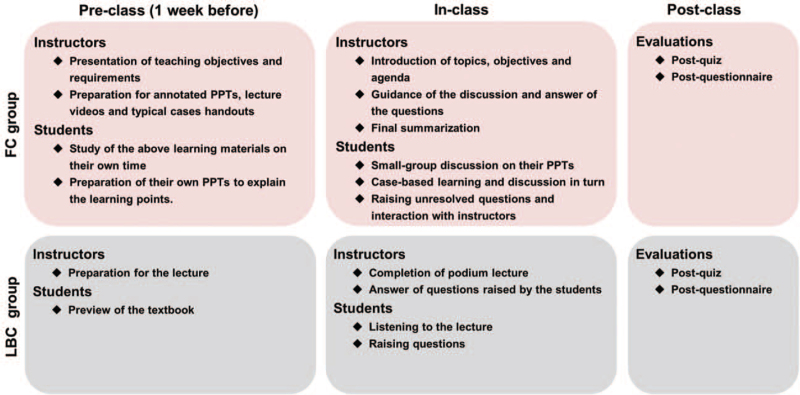
Graphical illustration of the teaching process in flipped classroom modality and lecture-based classroom modality.

The ninth edition of Pathology published by People's Medical Publishing House was used for pathology teaching. Two sections in the textbook (cardiovascular and respiratory system diseases) were chosen to implement FC combined with CBL teaching modality in this study, with a total of 12 class hours. The FC process was administered according to the guidelines described by Yang with minor modifications.^[18]^ The study was performed complying with the flowchart as stated in Figure 1. In the pre-class section of FC, the instructor briefed students on the FC model and provided study materials on the course website one week before the class. The course materials included learning purposes and requirements, tutor-generated annotated PowerPoints (PPTs), web-based video lectures and typical case handouts. Participants were asked to study the learning materials on their own time and prepare their PPTs to explain the learning points. The in-class session started with a brief outline of lectures by the instructor, followed by a brief presentation and discussion on students’ own PPTs within pre-assigned groups (n = 7–8). Students in groups then collaborated to take turns interpreting and discussing the real clinical cases proposed by instructors. These cases of cardiovascular or respiratory system diseases were not disclosed to students until the class convened. The instructor provided guidance and feedback during the progress of students’ interpretations and indicated the feature and atypical findings for each case. Finally, the instructor summarized the concepts and went over the arduous questions raised by students. In the traditional LBC, students were encouraged to preview the textbook before the class and attended a didactic lecture carried out by the instructor. A traditional question-and-answer session was included in LBC class. The classes in FC or LBC were conducted by the same instructor to guarantee the consistency of the teaching content and objectives in the 2 teaching approaches.

### 2.3 Data evaluation

**Figure 2:**
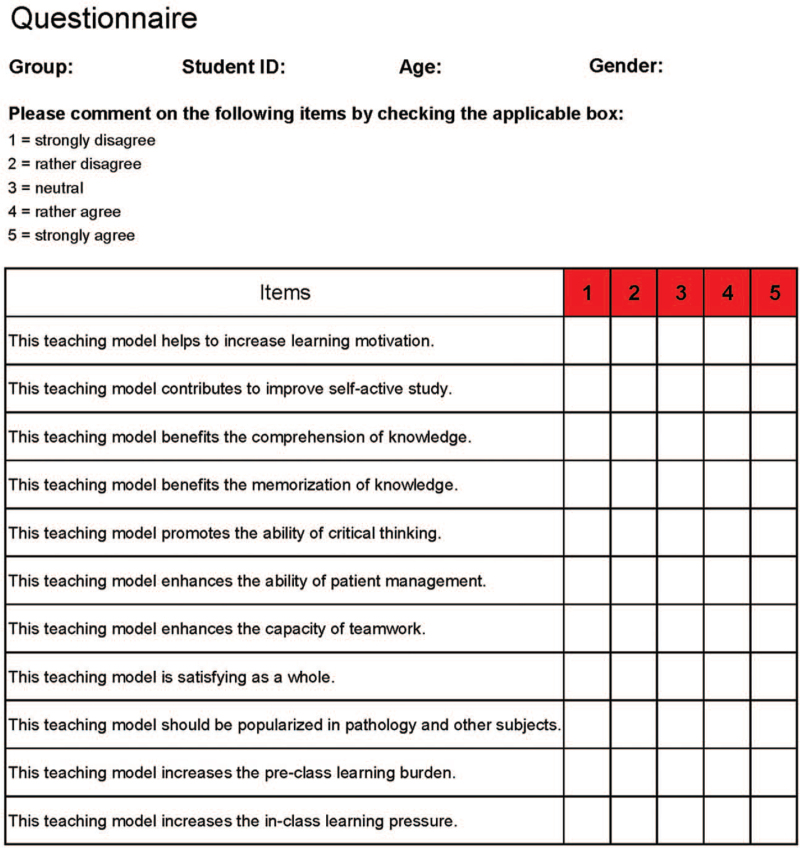
The evaluation sheet applied in this study.

When completing each section of teaching content, students in both the FC and LBC groups were asked to enter a post-class quiz to evaluate their learning outcomes. All items in the post-class quiz were A2-type questions proposed to evaluate students’ mastery of basic theoretical knowledge and students’ clinical case analysis ability. Based on Bloom's taxonomy of cognitive learning objectives,^[19,20]^ the categories of “remember” and “understand” collapsed into “basic theoretical knowledge,” and items in other categories were regarded as “clinical case analysis”. Moreover, an online questionnaire using WeChat was applied to collect data on students’ feedback and perceptions of the 2 teaching models. The questionnaire was modified based on previous references with verified reliability and validity,^[21–23]^ and was composed of 11 Likert-type items covering both positive and negative aspects (Fig. 2), with a 5-point scale (1 = strongly disagree, 2 = rather disagree, 3 = neutral, 4 = rather agree, 5 = strongly agree).

### 2.4 Statistical analysis

Statistical analysis was performed by Statistical Product and Service Solutions (SPSS) 16.0 software (SPSS Company, Chicago, IL). Normal distribution and homogeneity of variance of the data were evaluated. The scores of 5-point Likert scale in the survey were compared between the 2 groups by nonparametric Mann–Whitney Test. The mid-term examination scores and post-quiz scores were analyzed by independent samples *t* test. The *χ*^2^ test was used to analyze the sex and nationality match. The statistical data were presented as mean ± standard error of the mean (SEM). *P* < .05 was considered statistically significant.

## 3 Results

### 3.1 Baseline characteristic

**Figure 3:**
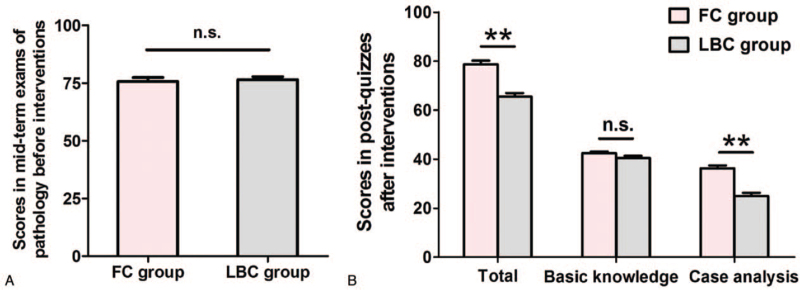
Comparison of students’ scores in mid-term examinations of pathology before interventions and in post-quizzes after interventions between the flipped classroom and lecture-based classroom groups. (A) Scores in the mid-term examinations before interventions. (B) Scores in the post-quizzes after interventions. Data are presented as mean ± SEM. Asterisks (∗) show the significance of the difference (2 tails): ^∗∗^*P* < .01 compared with LBC group. n.s. = not significant compared with LBC group.

A total of 117 students were assigned to the FC group (n = 59) or LBC group (n = 58). In Table 1, there was no significant difference between the FC group and the LBC group in terms of age (*P* = .753), sex (*P* = .775), and nationality (*P* = .662), suggesting a good demographic match between the 2 groups. The analysis of the mid-term examination scores before interventions was performed to evaluate whether the previous performance on pathology learning of the students from the 2 groups was comparable. In Figure 3A, no apparent difference in the mid-term examination scores of pathology between the FC and LBC groups (*P* = .718) indicated that students’ learning levels and abilities in the 2 groups were nearly equal.

**Table 1:** Demographic information of participants in this study.

	FC group (n = 59)	LBC group (n = 58)	*P*
Age (mean ± SEM)	20.19 ± 0.11	20.14 ± 0.11	.753
Sex			.775
Male (percentage)	31 (52.54%)	32 (55.17%)	
Female (percentage)	28 (47.46%)	26 (44.83%)	
Nationality			.662
Han (percentage)	56 (94.92%)	56 (96.55%)	
Others (percentage)	3 (5.08%)	2 (3.45%)	

FC = flipped classroom, LBC = lecture-based classroom.

### 3.2 Students’ performance in the post-class quizzes

The efficacy of the 2 teaching modalities was assessed by a post-class quiz, which was conducted when finishing the each section of teaching content. The response rates of students in both groups in 2 post-class quizzes were 100%. In Figure 3B, the students in the FC group gained higher scores (the average of 2 quizzes) in the post-class quizzes than those in the LBC group (78.73 ± 1.53 vs 65.52 ± 1.48, *P* < .001). Furthermore, the scores related to basic theoretical knowledge in the FC and LBC groups were 42.46 ± 0.66 and 40.52 ± 0.88, respectively, with no statistically significant difference (*P* = .089). However, higher scores regarding the questions of clinical case analysis were observed in the FC group than in the LBC group (36.27 ± 1.22 vs 25.01 ± 1.26, *P* < .001). Our findings revealed that both the FC and LBC models were suitable for passing on basic theoretical knowledge, whereas the FC model exhibited greater advantages than the LBC model in developing the higher level of cognitive abilities.

### 3.3 Students’ self-perceived competence and opinions in the questionnaires

**Figure 4:**
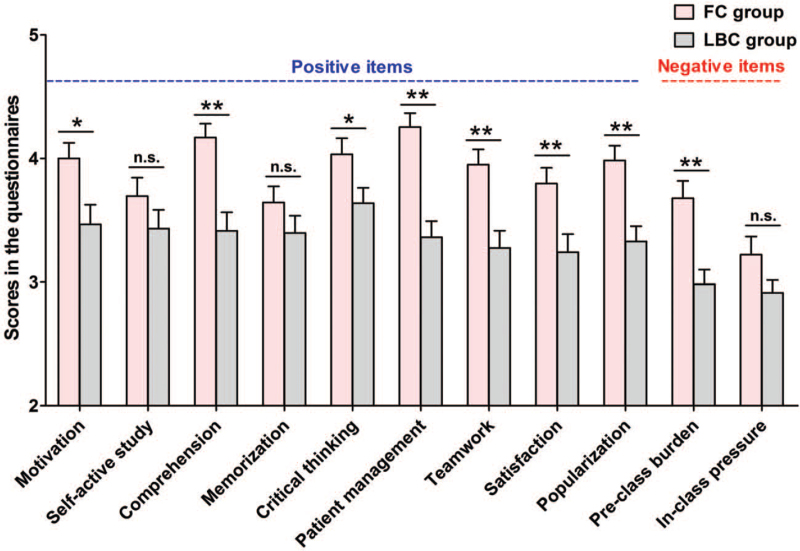
Comparison of students’ self-perceived competence and opinions on the teaching model between the flipped classroom and lecture-based classroom groups. Data are presented as mean ± SEM. Asterisks (∗) show the significance of the difference (2 tails): ^∗^*P* < .05, ^∗∗^*P* < .01 compared with LBC group. n.s. = not significant compared with LBC group.

Students who participated in this study finished the online questionnaires on their self-perceived competence and opinions towards FC or LBC teaching modality. The response rates of students in both groups for the questionnaires were 100%. In Figure 4, compared with LBC, more students believed that the FC approach improved their learning motivation (*P* = .019), with no difference in increasing self-active study (*P* = .208). In addition, the FC model was believed to significantly enhance the students’ abilities of comprehension of knowledge (*P* < .001), critical thinking (*P* = .024), and patient management (*P* < .001) compared with the LBC model, but failed to benefit the memorization of fundamental knowledge (*P* = .183). These results were consistent with students’ performance in post-quizzes, where FC modality significantly raised students’ scores in higher levels of cognitive abilities (ie, the ability of clinical case analysis), rather than scores in basic theoretical knowledge. Moreover, this study revealed that FC significantly improved students’ teamwork compared with LBC (*P* < .001). The positive responses led to a higher rate of satisfaction with FC than LBC (*P* = .006), and students agreed that FC rather than LBC should be popularized in pathology and other subjects (*P* < .001). For negative items, more students agreed that FC increased pre-class burden than LBC (*P* < .001), whereas no significant difference was found in the students’ opinion on in-class pressure between the 2 groups (*P* = 0.116).

## 4 Discussion

With the rapid development of medical and science techniques, the traditional model of pathology education cannot fulfill the needs of current medical education systems.^[3]^ Recently, there is a shift in education methodology from traditional teacher-centered didactic lectures to student-centered active learning approaches, including FC and CBL, which are becoming increasingly popular in medical education.^[7,16,18]^ In this study, we implemented FC combined with CBL in the teaching of pathology, where students were required to study preprovided course materials pre-class, followed by clinical case-based interactive group discussion in-class. To date, this innovative modality has not been well examined in undergraduate pathology education. We compared students’ performance and perceptions of this format with those of the traditional LBC teaching model. We found that students preferred the CBL-based FC modality as a whole, which can realize all-round teaching aims and promote students’ various cardinal skills.

We explored the effectiveness of FC combined with CBL in the pathology education of undergraduate medical students. Here, we revealed that the scores in post-class quizzes in the FC group were much higher than those in the LBC group, which was mainly attributed to the increase in scores on case-analysis type of questions, but not theoretical knowledge-related questions. This suggests that although both FC and LBC may improve the acquisition of knowledge, the former makes students better understand and apply the new knowledge. Our findings were consistent with previous studies that the FC model applied in other medical subjects fostered students’ abilities in analyzing and solving clinical problems, thus improving the higher level of cognitive abilities.^[18,23,24]^ Multiple factors in the preparation and implementation of FC may contribute to this advantage of FC.^[7,25]^ In personalized pre-class studies, students in the FC group can arrange their self-paced study plans and learn poorly mastered knowledge multiple times. In in-class studies, students are encouraged to use what they learned pre-class to solve clinical problems during group discussions. Apart from simple knowledge mastery, this output process of FC emphasizes to foster the abilities of application, analysis and synthesis. Therefore, the FC model bridges the gap between pre-class knowledge learning and in-class cultivation of analyzing and solving abilities, and helps to connect theory to practice much better.

We then compared students’ cognitions and opinions on the FC model with those on the LBC model. Herein, stronger learning motivation was considered by students from FC group than LBC group, but no apparent difference in students’ opinion on self-active study. To perform well in group discussion in-class, students often have a better motivation for studying the pre-provided course materials and searching for additional web materials in pre-class study.^[26]^ However, since it is hard to implement supervision and management in students’ pre-class study,^[8]^ the consensus of self-active study can be diverse from person to person, thus causing students’ different performance in group discussion. Our findings suggest that instructors should convey the intention, value and implementation of the FC model in detail to all students before class and truly inspire students’ enthusiasm for self-active study without strict supervision from teachers. Additionally, FC was believed to be helpful to improve students’ abilities of knowledge comprehension, critical thinking and patient management, as reflected in students’ performance in the post-class quizzes where students in the FC group gained more scores than students in the LBC group, especially the scores in case-analysis type of questions. These findings are similar to previous studies that the FC approach can produce greater learning gains than the LBC model in many medical subjects, such as pharmacology, radiology, and anatomy.^[8,23,27]^ Furthermore, an improvement of students’ teamwork ability was found with the application of FC. Compared to LBC where there is only teacher-student interaction, the in-class study in FC using not only teacher–student interaction but also student-student interaction may contribute to promoting the teamwork ability, which is an essential ability to patient management for medical students.^[28]^ Overall, more students from FC group felt satisfaction with the teaching model than those from LBC group, and it was agreed that FC model should be popularized in the entire teaching of pathology since FC improved their wide-spectrum cognitive abilities.

However, although FC had the above advantages over LBC in teaching pathology, students in the FC group held the view that the pre-class study took up an amount of their spare time and gave negative feedback on the pre-class burden in FC. This negative feedback might be attributed to the following factors. First, compared to LBC, wherein students may spend more time after class to review and do homework, FC participants mainly perform their study in pre-class and in-class time. FC as a student-centered and active learning method requires additional time for self-study and preparation for in-class presentation and discussion. Second, since the traditional teacher-centered teaching method has been accustomed by students, the participants who had not previously participated in FC are unfamiliar with this novel teaching approach and do not know how to effectively learn in pre-class study. We speculate that this negative feedback on pre-class burden may be partially relieved with the adaptation to FC modality and prolonged time for pre-class study (eg, 2 weeks). It is well known that group discussion in-class as a vital element of FC modality often brings more in-class pressure, which might be a drawback of FC reported in previous studies.^[29,30]^ Intriguingly, students’ opinion on in-class pressure is not significantly changed with the application of FC combined with CBL. This phenomenon may be due to the usage of high-yield clinical cases, because the small group study via case-based discussions has been shown to provide a nonintimidating, interactive and supportive environment to foster students’ clinical reasoning and increase the overall enjoyment of learning.^[10,16]^ In this study, similar to previous findings,^[16]^ we believed that the students who took good advantage of preprovided course materials would participate in the case-based discussions with more confidence and less anxiety, thus causing no raised in-class pressure.

## 5 Limitations

Several limitations need to be considered. First, this study was conducted for a relatively small cohort of participants. Studies with more participants enrolled in may help to further verify the effectiveness and advantages of FC in pathology teaching. Second, the application of FC usually consumes generous human, material and financial resources. We spent more faculty time in preparation of learning resources, including pre-recorded video, annotated PPTs and typical clinical cases, as is required for FC teaching modality. For the above reason, we selected only 2 sections in the pathology textbook for the implementation of FC in this pilot study. Full preparation, overall consideration and careful planning are needed before applying this teaching model in the entire teaching of pathology. Third, we focused on pre-class and in-class activities, but did not extend the study to after-class activities, which are conducive to consolidating the prior learned knowledge by continuous practice. The after-class activities can be achieved in a structured manner through additional programs in future.

## 6 Conclusions

In conclusion, our findings suggest that FC combined with CBL as a promising and effective modality may be helpful to improve students’ performance and promote their multiple cardinal skills during undergraduate pathology education. Further optimizations in course design, course management and course evaluation can fulfill the application of this innovative approach in pathology and other medicine curricula in medical colleges.

## Author contributions

**Conceptualization:** Li Cai, Rong Li.

**Data curation:** Li Cai, Rong Li.

**Funding acquisition:** Li Cai.

**Investigation:** Li Cai, Yan-li Li, Xiang-yang Hu.

**Writing – original draft:** Yan-li Li.

**Writing – review & editing:** Li Cai.
